# Survival outcome of different treatment sequences in patients with locally advanced and metastatic pancreatic cancer

**DOI:** 10.1186/s12885-024-11823-8

**Published:** 2024-01-12

**Authors:** Mengjiao Fan, Guochao Deng, Yue Ma, Haiyan Si, Zhikuan Wang, Guanghai Dai

**Affiliations:** 1 Medical School of Chinese People’s Liberation Army, Beijing, China; 2 Department of oncology, The Fifth Medical Center, Chinese People’s Liberation Army General Hospital, Beijing, China; 3 Department of oncology, The First Medical Center, Chinese People’s Liberation Army General Hospital, Beijing, China

**Keywords:** Pancreatic ductal adenocarcinoma, Treatment landscape, Treatment patterns, Treatment sequences, New chemotherapy regimens, Irinotecan, Nab-paclitaxel, FOLFIRINOX

## Abstract

**Background:**

Despite some therapeutic advances, improvement in survival rates of unresectable and/or metastatic pancreatic ductal adenocarcinoma (PDAC) has been minimal over recent decade. We aimed to evaluate the impact of different treatment sequences on clinical outcomes of advanced PDAC at our academic institution.

**Methods:**

In this single institution retrospective analysis, we assessed characteristics and survival rates of unresectable and/or metastatic pancreatic PDAC patients who started a systemic treatment between 01/2015 and 12/2021. Survival analyses were performed by Kaplan-Meier and Cox proportional hazards model.

**Results:**

The number of 285 patients received at least two lines of treatment, but only 137 patients were suitable for third-line treatment. Subgroup analysis showed that thirty-seven patients received A line (gemcitabine/nab-paclitaxel or nab-paclitaxel combined therapy to FOLFIRINOX) therapy, 37 patients received B line (nab-paclitaxel combined therapy to gemcitabine combined therapy to FOLFIRINOX) therapy, 21 patients received C line (nab-paclitaxel combined therapy to gemcitabine combined therapy to oxaliplatin or irinotecan combined therapy) therapy. Survival rates for different treatment lines were significantly different and median overall survival (OS) was 14.00, 18.00, and 14.00 months, respectively (p<0.05).

**Conclusion:**

Our study provides real-world evidence for the effectiveness of different treatment sequences and underscores the treatment sequences on survival outcome when considering the entire management in advanced PDAC.

**Supplementary Information:**

The online version contains supplementary material available at 10.1186/s12885-024-11823-8.

## Introduction

With a persistently increasing incidence and minimal change in mortality rates, Pancreatic ductal adenocarcinoma (PDAC) will become the second leading cause of cancer-related mortality by 2030, trailing only lung cancer [1]. By far, the cause of PDAC has not been well understood, and some factors, such as advancing age, family history, smoking and alcohol abuse, male, diabetes and obesity has been associated with PDAC [2]. One reason for its poor prognosis is that most patients are diagnosed at a metastatic or locally advanced disease stage [3]. In addition, although roughly 50% of patients with PDAC have no clinically detectable metastases at presentation, early relapse and limited efficacy of available drugs also lead to an extremely poor prognosis [4].

Systemic chemotherapy remains the standard treatment of metastatic PDAC. After years of limited therapeutic progress in advanced PDAC, characterized by one negative phase III study after another, we have gained some available therapeutic options for this disease. In the past, gemcitabine (Gem) monotherapy was the only approved first-line treatment in patients with advanced PDAC [5]. After then, many different Gem-based combinations have been clinically tested. More recently, the PRODIGE4/ACCORD11 study showed promising effects treated by FOLFIRINOX (the combination of 5-FU, leucovorin (LV), irinotecan, and oxaliplatin) and the MPACT trails also demonstrated superior survival with the combination of Gem plus nab-paclitaxel (NabP/Gem) over Gem in metastatic PDAC [6–7]. As an oral fluropyrimidine, S-1 has been demonstrated its effectiveness and less adverse events in the treatment of postoperative PDAC compared to Gem [8–9]. Meanwhile, a phase II study has demonstrated the efficacy and safety of NabP in combination with S-1 as the first-line treatment in patients with locally advanced and metastatic PDAC. After then, the combination treatment with nanoliposomal irinotecan (nal-IRI) and 5-fluorouracil/leucovorin(5-FU/LV) has become the first approval of a second-line treatment option for patients with advanced PDAC who have been previously treated with Gem-based chemotherapy [10].

Beyond classical cytotoxic agents, a variety of therapeutic approaches have been shown to improve the outlook of patients with advanced PDAC. Except for KRAS signal inhibitor and stromal-depleting agents, immunotherapy approaches, most notably immunocheckpoint inhibitors (ICI), have demonstrated efficacy in a variety of solid tumors. PDAC, however, has generally been considered a nonimmunogenic malignancy, insofar as tumor-infiltrating effector T lymphocytes do not represent a histopathologic hallmark of this disease [11–12].

Despite these advances, current standard of care treatments only led to a 5-year survival rate of about 10% in all PDAC patients and only 1% in the case of advanced disease [2–3]. Furthermore, improvement in survival rates of unresectable and/or metastatic disease has been minimal over recent decade [13–14]. FOLFIRINOX and NabP/Gem were equally effective as first-line treatment as reported by previous studies, but there is a lack of clinical studies with direct head-to-head comparisons. Ursula M. Vog showed that the sequence of these two regimens did not influence overall survival (OS), and both groups had a median survival of approximately 14 months. Moreover, they also indicated that second-line treatment after NabPGem with FOLFIRINOX is possible and effective in a considerable number of patients. One years later, Markus Kieler demonstrated that survival rates for different first to second line treatment sequences (modified FOLFIRINOX to NabP/Gem, NabP/Gem to nal-IRI, or NabP/Gem to fluoropyrimidines plus oxaliplatin (FOLFOX) were not significantly different and median OS ranged from 14.27 to 15.64 months.

In this retrospective analysis, we reported the outcome of patients with advanced PDAC who received different treatment lines in our institution over the past 7 years.

## Methods

### Study design

This is a single-center, retrospective, observational study, including patients with histologically or cytologically proven non-resectable PDAC which was either locally advanced or metastasized and who have started a systemic treatment at the First and Five medical center of the PLA general hospital of China between 01/2015 and 12/2021.

Gemcitabine was administered with 1000 mg/m^2^ after application of nab-paclitaxel (125 mg/m^2^) on days 1 and 8, every 21 days. S-1 was given twice a dayorally at a dose according to the body surface area (BSA) (< 1.25 m^2^, 80 mg/d; ≥ 1.25 to < 1.5 m^2^, 100 mg/d; ≥1.5 m^2^, 120 mg/d) on days 1 through 14 of each 21-day cycle. FOLFIRINOX (oxaliplatin, 60–65 mg/m^2^ on days 1; irinotecan, 120–135 mg/m^2^ on days 1; leucovorin, 400 mg/m^2^ on days 1; and fluorouracil, 400 mg/m^2^ given as a bolus followed by 1200-1600 mg per square meter given as a 46-hour continuous infusion, every 2 weeks) was given as described. In the event of adverse events, dose reduction and/or delay and drug secondary prevention were selected based on the discretion of the physician. The health care system of the PLA general hospital is powerful and all data are completely saved at the hospital’s big data management center. The digital health care system PRIDE was used to identify eligible patients and then the big data center counted the patient lists. Inclusion criteria were: male and female, at least 18 years age, diagnosed PDAC, registration at any of medical center, and the initiation of first-line treatment between 01/2015 and 12/2021. Exclusion criteria were histopathology other than adenocarcinoma or secondary metastatic tumor of non-pancreatic origin and patients participating in industry funded trails. The selection of the optimal treatment options was based on the at that time available national and international treatment guidelines.

For the comparison of the main study cohorts, the time of first administration of systemic chemotherapy, the time of disease progression and the time of death date were retrieved. The electronic medical history was queried for patient information, disease statues, treatment details and overall survival. The survival time was judged by telephone and the corresponding date nodes were recorded. The data involved in this article have been approved by the ethics Committee of PLA General Hospital, and all patients signed informed consent before therapy and was performed according to the Helsinki criteria for good scientific practice.

### Statistics

SPSS22.0 software was used to organize and analyze the data. Descriptives were calculated as median frequencies and percentages. Chi-square test was used for statistical comparison of categorical variables, and unpaired t-test or univariate ANOVA test were used for comparison of metric variables. Overall survival (OS) and progression-free survival (PFS) were calculated from the date of the treatment initiation until the date of death or the date of documentation of disease progression or death in patients without disease progression, whichever occurred first. Multivariate survival analysis was performed using Kaplan Meier plot, log-rank test, and Cox proportional hazards model. A *P* value of 0.05 was considered statistically significant. GraphPad Prism Software was the drawing software.

## Results

### Patients and treatment

From January 2015 to December 2021, 403 patients were screened and 285 patients received at least two lines of treatment. Two-hundred and seventy-six (*n* = 274) patients (97%) had metastatic disease and 11 patients (4%) had locally advanced PDAC. Specific gene states, including BRAC mutation, KRAS wild type, and MSI-H were excluded in this study. The median follow-up time was 398 days (range from 78 days to 1230 days). Of all the patients, 256 events occurred (90%) and the median OS was 12.0 months.

### Baseline characteristics of patients are shown in Table 1

The median age was 56 years (range 30–78), and 61% were male. Most of the patients presented with good Eastern Cooperative Oncology Group (ECOG) performance status (47% with ECOG 0 and 48% with ECOG 1) and 5% had an ECOG performance status of 2. At the time of initiation of systemic chemotherapy, 11 patients (4%) had locally advanced, inoperable disease. The majority of the metastatic disease sites were in the liver (80%), in the peritoneal (12%) and in the lymph node (39%). 52% of the patients had more than one metastatic site at the time of receiving first line treatment. Baseline levels of CA199 were significantly elevated in 77% of all patients. A small proportion of patients had a negative CA199 at baseline.

**Table 1:** Baseline characteristics of patients

Characteristic	Total *n* = 285	%
Median age(range)	56	(30–78)
Sex		
male	174	61
female	111	39
Age		
under 55 years	127	45
over 55 years	158	55
ECOG grade		
0	133	47
1	135	48
2	17	5
Diabetes		
0	220	77
1	65	23
Smoke		
0	190	67
1	95	33
Drink		
0	225	79
1	60	21
Number of metastatic sites		
0	11	4
1	125	44
More than 1	149	52
Liver metastasis		
0	57	20
1	228	80
Peritoneum metastasis		
0	199	88
1	86	12
Lymph node metastatic		
0	173	61
1	112	39
CA199		
Normal	65	23
Abnormal	220	77
CA199		
Lower than 1000	180	63
More than 1000	105	37
Jaundice		
0	249	87
1	36	13

As shown in Table 2, in the first-line systemic therapy, NabP plus S-1 (NabP/S-1) and NabP/Gem rank on top. One hundred and sixty-six patients (58%) were treated with NabP/S-1 and twenty-three patients (8%) were treated with NabP/Gem. Further commonly used regimens were FOLFIRINOX and Gem alone therapy. In the second-line treatment, Gem combined therapy were the most commonly used regimens (136 patients, 48%). Administered therapies were FOLFIRINOX (28 patients, 10%), NabP combined therapy (54 patients, 19%), Irinotecan combination therapy (9 patients, 3%), Gem alone (19 patients, 7%).

The proportion of patients which started a third-line therapy was 48% (*n* = 137). These therapies were FOLFIRINOX (43 patients, 31%), immunotherapy (26 patients, 18%), Gem combined therapy (14 patients, 10%), irinotecan or oxaliplatin combined therapy (31 patients, 23%) and others (23 patients, 17%).

**Table 2:** Chemotherapy regimens received

Chemotherapy regimens received	N	%
First-line	285	100
NabP/S-1	166	58
NabP/Gem	23	8
Gem/Oxaliplatin	16	6
FOLFIRINOX	28	10
Gem alone	19	7
Other therapies	33	12
Second-line therapy	285	100
Gem combined therapy	136	48
FOLFIRINOX	28	10
Gem alone	19	7
NabP combined therapy	54	19
Other therapies	48	17
Death after 2nd-line or not suitable for 3rd-line	148	52
Third-line treatment	137	100
FOLFIRINOX	43	31
Immunotherapy	26	19
Gem combined therapy	14	10
Irinotecan or Oxaliplatin combined therapy	31	23
Other therapies	23	17
Fourth-line therapy	30	100

### Systemic chemotherapy: efficacy of treatment landscape

One hundred and thirty-seven (*n* = 137, 48%) received third-line therapy in this study. We found that NabP combination therapy in first-line, Gem combination therapy in second-line, or cross, (153 patients, 77 of whom received third-line chemotherapy and 76 patients did not receive third-line chemotherapy) was comparable to patients receiving NabP/Gem (37 patients) in first-line and FOLFIRINOX in second-line (mOS: 13.00 versus 14.00 months, *p* = 0.613, log rank).

Based on these data, we further analyzed the differences in survival outcomes among different treatment sequences. A total number of 95 patients receiving at least two lines of therapy (Systemic chemotherapy should include standard or modified treatment regimen) were included in the further analysis. A line, 37 patients, refers to NabP/Gem or NabP combined therapy to FOLFIRINOX (red line), B line, 37 patients, refers to NabP combined therapy to Gem combined therapy to FOLFIRINOX, (green line) and C line, 21 patients, refers to NabP combined therapy to Gem combined therapy to oxaliplatin or irinotecan combined therapy (purple line).

The patient characteristics of the three lines are summarized in Table 3. The median age of three line was A line 55 years (range 33–68 years), B line 53 years (range 30–70 years), and C line 56 years (range 44–68 years). Of three lines, 59% were male in A line, 62% were male in B line, and 62% were male in C line. At the time of initiation of systemic chemotherapy, 1 patient in line A, 1 patient in line B, and 2 patients in line C had locally advanced, inoperable disease. Most patients presented with metastatic disease before systemic therapy. Only a small proportion of patients had a negative CA199 at baseline (A line 24%, B line 30% and C line 23%). The majority of the metastatic disease sites were liver. Around 50% of patients had more than one metastatic site at the time of receiving first line treatment (A line 52%, B line 51%, and C line 67%). Most of the patients presented with good ECOG performance status (A line 94%, B line 93% and C line 95% with ECOG 0–1).

**Table 3:** The patient characteristics of three lines

Characteristic	A *n* = 37	B *n* = 37	C *n* = 21	n	*P* value
Sex					1.000
Female	22	23	13	58	
Male	15	14	8	37	
Age (year)	55.38	53.32	56.10	57.74	0.176
Age					0.836
under 55 years	18	19	12	49	
over 55 years	19	18	9	46	
ECOG score					0.961
0	15	16	8	39	
1	20	18	12	50	
2	2	3	1	6	
Diabetes					0.926
0	33	32	19	84	
1	4	5	2	11	
Smoke					0.632
0	27	29	14	70	
1	10	8	7	25	
Drink					0.300
0	28	32	19	79	
1	9	5	2	16	
Jaundice					0.428
0	32	35	18	85	
1	5	2	3	10	
Number of metastatic sites					0.482
0	1	1	2	4	
1	17	18	5	40	
More than 1	19	18	14	51	
Liver metastasis					0419
0	9	7	2	18	
1	28	30	19	77	
Peritoneum metastasis					0.816
0	25	26	13	64	
1	12	11	8	31	
Lymph node metastatic					0.890
0	24	22	14	60	
1	13	15	7	35	
CA199					0.871
Normal	9	11	5	25	
Abnormal	28	26	16	70	
Primary site					0.357
Head, body, or head and body	20	26	13	59	
Tail	17	11	8	36	
Ampulla	0	0	0	0	

**Fig. 1:**
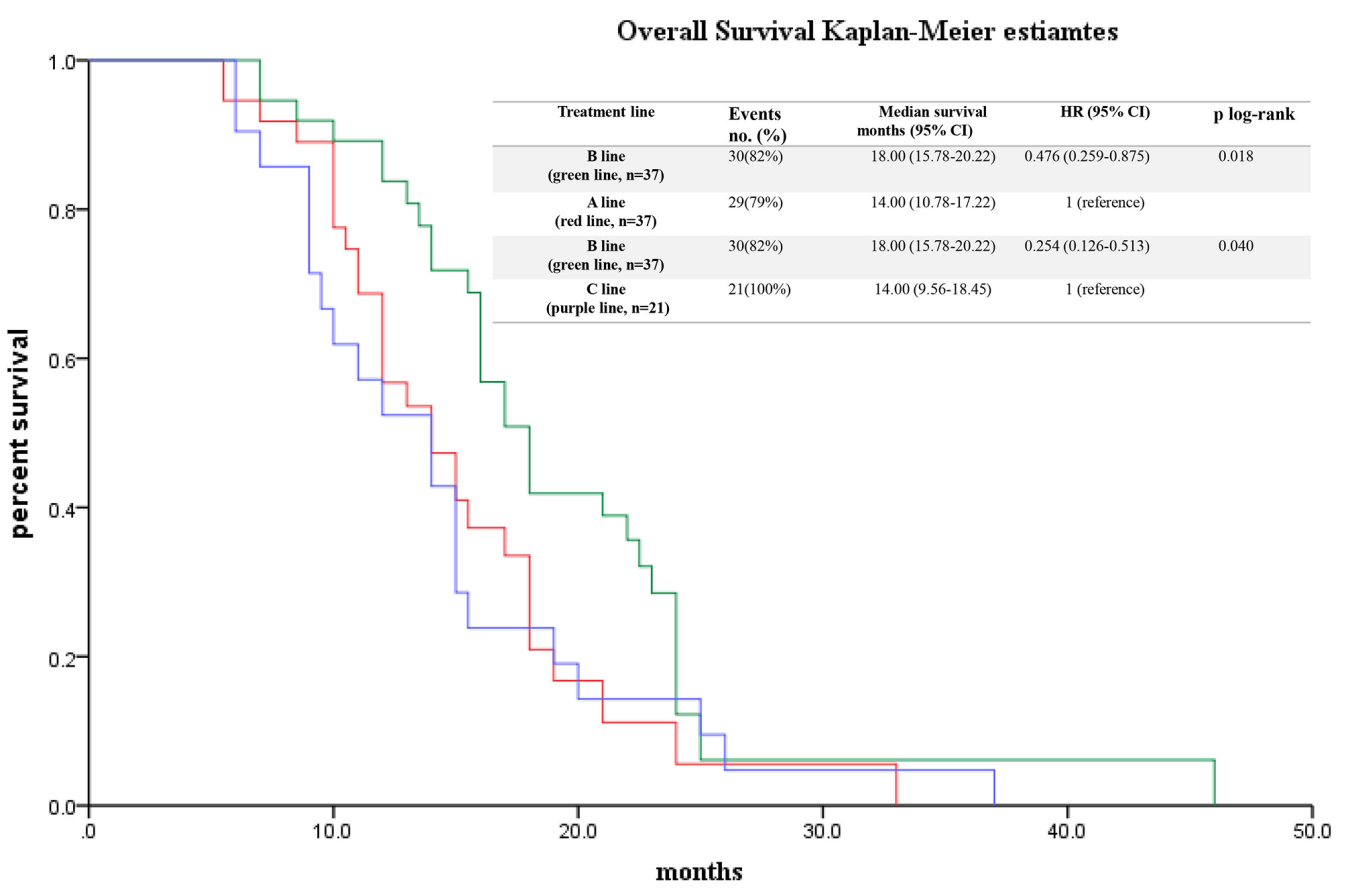
Survival of different treatment sequences. Legends: Kaplan-Meier curves of patients who were treated with one of the three treatment sequences from first to second and third line: A line, 37 patients, refers to NabP/Gem or NabP combined therapy to FOLFIRINOX (red line), B line, 37 patients, refers to NabP combined therapy to Gem combined therapy to FOLFIRINOX (green line) and C line, 21 patients, refers to NabP combined therapy to Gem combined therapy to oxaliplatin or irinotecan combined therapy (purple line)

**Fig. 2:**
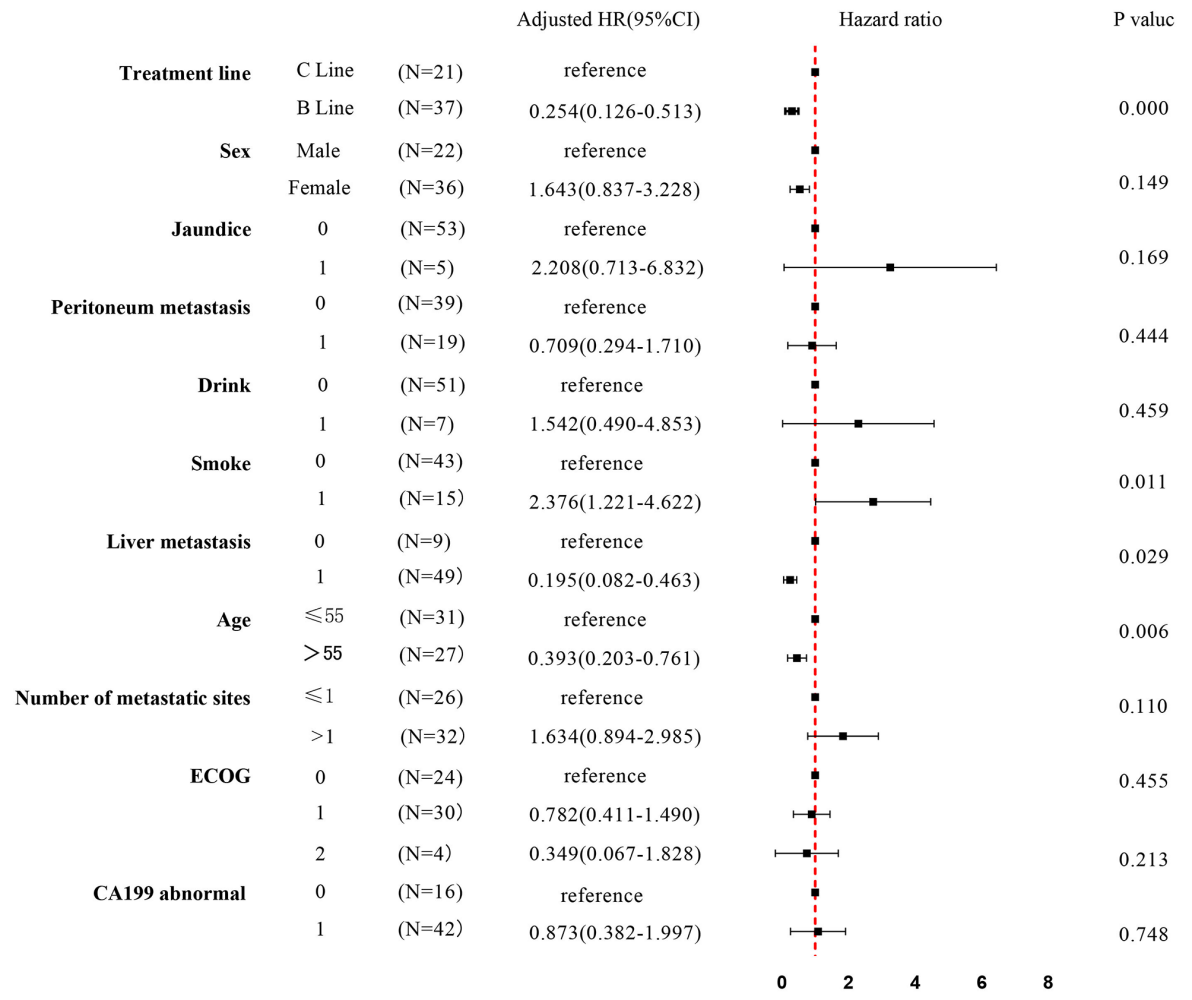
Subgroup analysis by forest plot, with HRs for OS, in the patients treated with B line and C line. Abbreviations: ECOG, Eastern Cooperative Oncology Group performance scale; B line, NabP combined therapy to Gem combined therapy to FOLFIRINOX; C line, NabP combined therapy to Gem combined therapy to oxaliplatin or irinotecan combined therapy

**Fig. 3:**
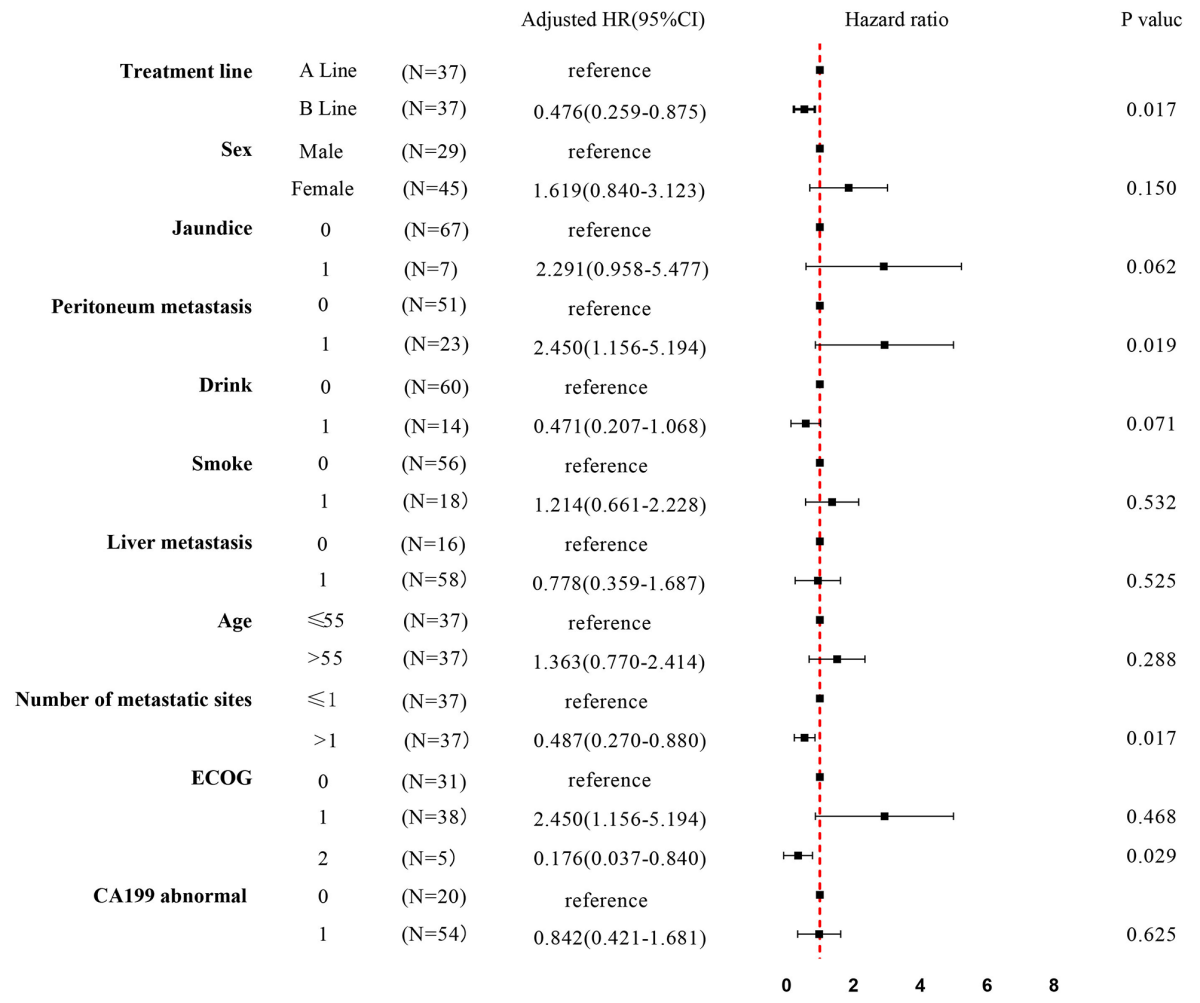
Subgroup analysis by forest plot, with HRs for OS, in the patients treated with A line and B line. Abbreviations: ECOG, Eastern Cooperative Oncology Group performance scale; A line, NabP/Gem or NabP combined therapy to FOLFIRINOX; B line, NabP combined therapy to Gem combined therapy to FOLFIRINOX

The impact of different treatment sequences on survival outcome is presented in Fig. 1. The median OS of these three lines was that A line was 14.00 ± 1.64 months, 95% CI 10.78–17.22 months; B line 18.00 ± 1.13 months, 95% CI 15.78–20.22 months; and C line 14.00 ± 2.26 months, 95% CI 9.56–18.45 months (*P* = 0.042, log rank). Survival analysis (Cox proportional hazard model) with potentially influencing variables was performed for these three lines (Figs. 2 and 3). Among the subgroup, three different first to second and third line treatment sequences were analyzed. B line (green line, NabP combined therapy to Gem combined therapy to FOLFIRINOX) (mOS 18.00 months) differ significantly from C line (purple line, NabP combined therapy to Gem combined therapy to oxaliplatin or irinotecan combined therapy) (mOS 14.00 months) (*p* = 0.000, HR = 0.254, 0.126–0.513); B line (green line, NabP combined therapy to Gem combined therapy to FOLFIRINOX) (mOS 18.00 months) differ significantly from A line (red line, NabP/Gem or NabP combined therapy to FOLFIRINOX) (mOS 14.00 months) (*p* = 0.017, HR = 0.476, 0.259–0.875).

### Systemic chemotherapy: toxicity

In this study, FOLFIRINOX was applied to 74 patients mainly as second-line and third-line treatment. But due to reduced performance status or expected drug toxicity, all patients need a dose reduction (oxaliplatin, 60–65 mg/m^2^ on days 1; irinotecan, 120–135 mg/m^2^ on days 1; leucovorin, 400 mg/m^2^ on days 1; and fluorouracil, 400 mg/m^2^ given as a bolus followed by 1200-1600 mg per square meter given as a 46-hour continuous infusion, every 2 weeks). We observed the PFS of 5.5 months(5.0 months in the second-line and 6.0 months in the third-line)in the FOLFIRINOX dose reduction group with good tolerability. Adverse events of grade 3 and higher were experienced by 5 patients (14%) in the second-line and 4 patients (11%) in the third-line treatment. The most frequent side effects were neutropenia, fatigue and vomiting or nausea.

Patients receiving Gem/NabP, Gem combinations and NabP combinations also had a lower prevalence of 3 and higher adverse events, occurring in the 6 (16%) of Gem/NabP, 2 (5%) of Gem combinations and 2 (5%) of NabP combinations treated patients. Besides, the number of patients need dose reduction during the course of the treatment was 4 (11%) in the Gem/NabP group, 3 (8%) in the Gem combinations group and 2 (5%) in the NabP combinations group. Grade 3 or more neutropenia, thrombocytopeniaand and peripheral neurotoxicity were the main adverse events leading to a dose reduction, a change in treatment schedule or a lower quality of life.

## Discussion

There is limited evidence in the literature regarding the choice of first-line and the whole-line treatment for patients with advanced PDAC, which are highlighting the main findings of our study. We found that the completion rate of the third-line treatment caused by different front-line regimens was significantly different. The sequence of NabP combined therapy followed by Gem combined therapy followed by FOLFIRINOX led to the best OS outcome, which was encouraging.

We suggested that two major treatment strategies led to this outstanding OS data in our patient cohort. First, we offered novel chemotherapy lines (NabP combined therapy followed by Gem combined therapy followed by FOLFIRINOX) with continuous, toxicity-adapted treatment whenever performance status and patient preference allowed this strategy. Second, the choice of first-line treatment, as an effective treatment option, NabP/S-1, did not influence the decision to follow-up treatment plan. Therefore, a considerable number of patients were able to receive third-line chemotherapy and tolerate treatment toxicity with the most effective chemotherapy regimens known to date (Gem, NabP, and FOLFIRINOX) in PDAC [6, 13].

According to a review published in the journal of Cancer Treatment Reviews in 2021, both FOLFIRINOX and NabP/Gem regimens are feasible and comparable in the first-line setting [14]. FOLFIRINOX is preferred in the treatment of fit, young (< 65 years old) patients with few comorbidities and normal liver function, while NabP/Gem is used to treat less fit (ECOG PS: 1–2) and more vulnerable patients. Besides, a recent study evaluated the efficacy of NabP/Gem or FOLFIRINOX as first-line treatment in patients with unresectable PDAC [15]. Forty-two patients received FOLFIRINOX as first-line therapy, forty-one patients were treated with NabP/Gem as first line therapy. Forty-eight patients received both treatments. There was no significant difference in OS or PFS for either of the two sequences (*p* = 0.9). The OS for FOLFIRINOX followed by NabP/Gemor NabP/Gem followed by FOLFIRINOX was 13.7 months (95% CI: 12.6–14.7) and 13.8 months (95% CI: 8.6–19), respectively, which was similar with this study (NabP/Gem followed by FOLFIRINOX, mOS 14.00 months).

For many years, FOLFIRINOX was the first-line treatment of choice for patients with a good performance status. However, the percentage of patients with good performance status in the real-world population is usually significantly lower, especially in Asian populations. In our center, we treated approximately seventy of our patients between 2015 and 2021 with first-line NabP/S-1, and only a very small proportion with FOLFIRINOX. The efficacy and safety of NabP/S-1 as the first-line treatment in patients with locally advanced and metastatic PDAC have been demonstrated in previous study of our center and given these preclinical and preliminary clinical data, the combination of NabP/S-1 could theoretically be an option for PDAC [16, 17].

More than 40% of advanced PDAC can progress to receive second- or later-line chemotherapy [18]. It is difficult to choose a second-line treatment regimen for PDAC. Several factors, including performance status, drug availability, physician preference and prior first-line therapy affect treatment selection. Currently, five kinds of chemotherapeutic agents are recommended for patients withPDAC, including Five-Fluorouracil; nal-IRI, Gem, Oxaliplatin, and NabP. It is noteworthy that when some treatment in the first-line fails, the combination of FOLFIRINOX may represent a second-line treatment option, despite the lack of randomized clinical trials. Nonetheless, given that FOLFIRINOX is associated with a higher toxicity rate, FOLFIRINOX was not a preferred option in second-line treatment of our center. Furthermore, in the previously mentioned study by Kordes et al. patients who received FOLFIRINOX had a shorter median OS (9.9 months, 95% CI; 8.1–11.7) than previously reported [19]. In this study, we found that people who receiving FOLFIRINOX therapy can get less chances to receiving a third-line treatment when compared with gemcitabine combination therapy (38% vs. 55%). And this may be one reason for its poor OS. Treatment strategy for the third-line therapy in patients with metastatic PDAC is rarely rare, even patients have a relatively good performance state [14, 20]. It is remarkable that this study takes advantage of the first-line and second-line treatment and putting the FOLFIRINOX regimen, which has better survival outcome, into the third-line treatment.

The purpose of this article was to explore the whole-line treatment management to improve the survival outcome while increasing tolerance by separating NabP/Gem into NabP combination therapy and Gem combination therapy, or cross, reasonably. Previous studies have found that NabP combination therapy as first-line treatment for advanced PDAC showed good tolerability [16]. Regarding the study of treatment sequence, some previous studies have also given us a lot of insight [3, 21–26].Median OS of different treatment lines have been reported to be 9.7–19.1 months (Table 4). The outcomes of this study were comparable to previous reports.

**Table 4:** Previous studies on different treatment lines

Author	Year/design	Number of patients	Treatment lines	ORR (%)	DCR (%)	PFS (m)	OS (m)
Vogl, U et al. [21]	2019; retrospective	42; 41	FOLFIRINOX— NabP/Gem; NabP/Gem—FOLFIRINOX	43—9; 20—23	88—63; 74—77	PFS2: 3.2; PFS2: 5.7.	OS: 13.7 OS: 13.8 (*p* = 0.9)
Glassman, D.C et al. [22]	2018; retrospective	56	Gem based combinations—FP/Nal-IRI	5	46	PFS2: 2.9	OS2: 5.3
Kieler M et al. [3]	2020; retrospective	NA	FOLFIRINOX—NabP/Gem; NabP/Gem—FP/Nal-IRI; NabP/Gem—FP/Ox	NA	NA	NA	OS: 15.64; OS: 14.27; OS: 13.62. *p* = 0.068
Fukahori M et al. [23]	2023; retrospective	156; 77	FOLFIRINOX/NabP/Gem—Chmeotherapy; FOLFIRINOX/NabP/Gem ---BSC	NA	NA	NA	OS2: 5.2; OS2: 2.6.
Sütcüoğlu O et al. [24]	2023; retrospective	144	FOLFIRINOX—Gem based combinations	NA	NA	PFS2: 3.4	OS2: 6.7
Matsumoto T et al. [25]	2020; retrospective	23	NabP/Gem—FOLFIRINOX	23	68	PFS1: 5.3	OS: 12.1
Taieb J et al. [26]	2023; retrospective	263; 286; 228; 65; 32; 41	FOLFIRINOX—Gem based combinations; NabP/Gem—fluoropyrimidine combinations; FOLFIRINOX—Gem mono; NabP/Gem—fluoropyr mono; Gem mono—fluoropyrimidine combinations; Gem mono—fluoropyr mono.	NA	NA	NA	OS: 19.1; OS: 15.2; OS: 14.8; OS: 15.8; OS: 13.8; OS: 9.7
Present study	2023; retrospective	37; 37; 21	NabP/Gem— FOLFIRINOX; NabP based combinations—Gem based combinations—FOLFIRINOX; NabP based combinations—Gem based combinations— FP/Ox or FP /IRI	NA	NA	PFS1—PFS2—PFS3: 5.0—5.0—NA; 6.0—3.0—6.0; 5.0—2.0—3.5	OS: 14.0; OS: 18.0; OS: 14.0;

Abbreviations: (m)FOLFIRINOX, modified folinic acid, fluorouracil, irinotecan and oxaliplatin. Gem, gemcitabine. Mono, monotherapy. Nab-P, Nab-paclitaxel. PFS, progression free survival; PFS1, progression free survival of the first-line treatment; PFS2, progression free survival of the second-line treatment; PFS3, progression free survival of the third-line treatment; OS, overall survival; OS2, overall survival of the second-line; 5-FU, fluorouracil. Fluoropyr, fluoropyrimidine; ORR, objective response rate; DCR, disease control rate; FP/Nal-IRI, Nanoliposomal Irinotecan with fluorouracil; FP/Ox, Oxaliplatin with fluorouracil; NA, not applicable;

Besides, given that FOLFIRINOX is associated with a higher toxicity rate, this should be taken into account, especially with third-line treatment. Although the three-drug combination has a survival advantage over oxaliplatin combination or irinotecan combination in third-line treatment. Treatment had to be adapted since disease progression often leads to a rapid deterioration in patient ECOG and poor prognosis. Based on our study, the patients who planned to receive FOLFIRINOX as the third-line treatment and was de-escalated to FOLFIRI or FOLFOX duo to drug toxicity and poor ECOG score can still receive a survival benefit.

There were limitations in our study. This was a single-center, retrospective analysis. Another limitation of our study was that the changes in our clinical management may not well concern toxicities and dose adjustments during 2015 and 2020. However, this may not be very likely to affect the improved overall survival between our cohorts, as individual components of the different chemotherapies like Oxaliplatin, fluoropyrimidines, irinotecan, gemcitabine, as well as nab-paclitaxel are classic anti-tumor drugs which are available during this study.

To sum up, systemic chemotherapy remains the standard care for patients with metastatic PDAC, but progress is slow. Our study provides real-world evidence for the effectiveness of different treatment sequences and underscores the treatment sequences on survival outcome when considering the entire management in advanced PDAC.

### Electronic supplementary material

Below is the link to the electronic supplementary material.

Supplementary Material 1

## Data Availability

The data used to support the findings of this study are available from the corresponding author upon request.

## Abbreviations

PDAC: Pancreatic ductal adenocarcinoma
Gem: Gemcitabine
NabP: Nab-paclitaxel
NabP/Gem: Gemcitabine/nab-paclitaxel
FOLFIRINOX: The combination of 5-FU, leucovorin (LV), irinotecan, and oxaliplatin
Nal-IRI: Nanoliposomal irinotecan
Nal-IRI/5-FU/LV: The combination of 5-FU, leucovorin (LV), and nanoliposomal irinotecan
PFS: Progression-free survival
OS: Overall survival
ECOG: Eastern Cooperative Oncology Group
PS: Performance status
A line: Gemcitabine/nab-paclitaxel or nab-paclitaxel combined therapy to FOLFIRINOX
B line: Nab-paclitaxel combined therapy to gemcitabine combined therapy to FOLFIRINOX
C line: Nab-paclitaxel combined therapy to gemcitabine combined therapy to oxaliplatin or irinotecan combined therapy
