# Supplementary figures and images for: Survival outcome of different treatment sequences in patients with locally advanced and metastatic pancreatic cancer
Source: BMC Cancer. 2024 Jan 12;24:67. doi: 10.1186/s12885-024-11823-8 (PMC10785544; doi:10.1186/s12885-024-11823-8)

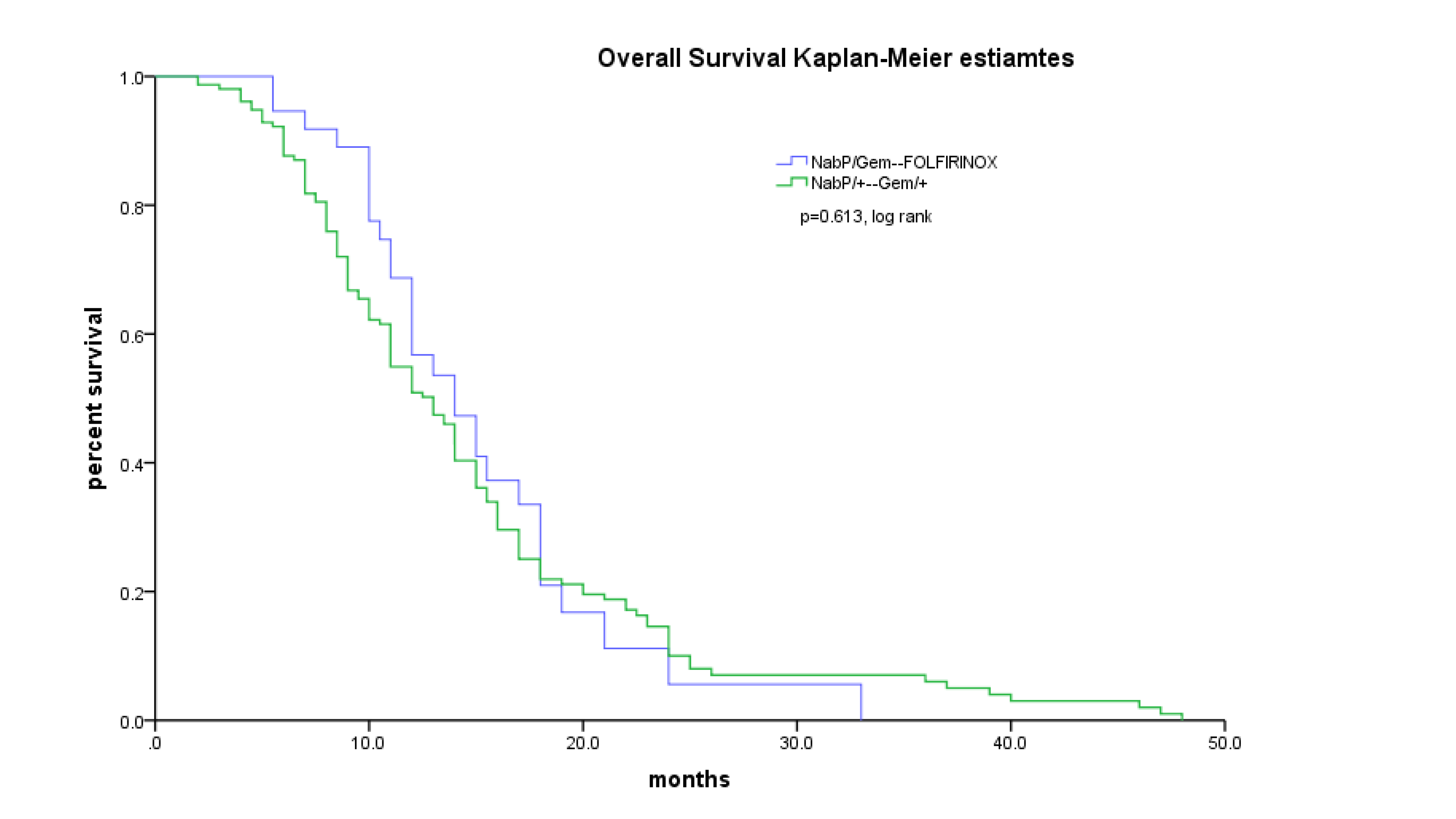

Supplement: Supplementary file 1 — Supplementary Material 1 [file 12885_2024_11823_MOESM1_ESM.jpg]
